# Numb Chin Syndrome as the Initial Presentation of Mandibular Metastasis of Colorectal Cancer: A Case Report

**DOI:** 10.7759/cureus.56133

**Published:** 2024-03-14

**Authors:** Yuichi Goto, Hiroshi Hijioka, Yoshinori Uchino, Tsuyoshi Sugiura, Tatsuo Okui

**Affiliations:** 1 Department of Maxillofacial Diagnostic and Surgical Science, Kagoshima University, Kagoshima, JPN; 2 Division of Oral and Maxillofacial Oncology and Surgical Science, Tohoku University, Sendai, JPN

**Keywords:** multiple metastases, mronj, mandibular metastasis, colorectal cancer, numb chin syndrome

## Abstract

Numb chin syndrome (NCS) is hypesthesia of the mandible and lower lip caused by damage to the inferior alveolar or mandibular nerves, commonly due to dental treatment or osteomyelitis, but occasionally caused by malignant tumors. We report the case of a male in his 60s. He came to our hospital with a chief complaint of mandibular pain and paresthesia in the right side of the mental region. He had noticed swelling of the left mandible one month before the initial visit and strong hypesthesia of the right side of the mental region one week before the initial visit. Panoramic radiographs showed slight osteosclerosis of the left side mandible at the initial visit. Blood tests showed only a slight inflammatory reaction. The diagnosis of mandibular osteomyelitis and numb chin syndrome was made, and a contrast-enhanced CT scan was performed to investigate the possibility of neoplastic lesions, but no obvious cause was found. Osteosclerosis was minimal. A tissue biopsy was recommended, but the patient did not consent. Considering the possibility of NCS due to a hematologic disorder, the patient was referred to a hematologist, but no cause could be identified at the initial visit. With time, the markedly severe pain worsened, and the possibility of a neoplastic lesion was again suspected. Blood tests were performed, which revealed abnormally high levels of CA19 and CEA. He consulted a gastroenterologist, who found a tumor in the ileocecal region on contrast-enhanced CT, and multiple systemic metastases were found on a PET-CT scan the next day. Systemic chemotherapy was administered for multiple metastatic unresectable colorectal cancer (cT4N1aMc2 stage IVc).

## Introduction

Numb chin syndrome (NCS) is a common cause of hypesthesia of the mandible and lower lip caused by damage to the inferior alveolar nerve or the mandibular nerve and is not limited to dental treatment or osteomyelitis. There have been scattered reports of mandibular metastases from hematologic diseases such as leukemia and malignant lymphoma, as well as prostate and breast cancer, in the case of solid tumors [1,2]. The number of patients with colorectal cancer has been increasing in recent years, and it is one of the most common cancers in the latest cancer statistics, with a high incidence reported in East Asia [3-5]. NCS as the first manifestation of cancer is extremely rare in the detection of mandibular metastases from colorectal cancer, compared to prostate or breast cancer. In this report, we describe a case of multiple metastases of colorectal cancer with NCS as the initial symptom, which was difficult to diagnose.

## Case presentation

A male patient in his 60s was diagnosed in May 2021 with complaints of pain in the right side of the mandible, sensory paralysis in the right side of the mentalist region, and swelling in the left mandible. The patient first noticed a mass sensation in the left side of the mandible around early April 2021, followed by a dull sensation in the right mentalist region a week before and pain in the right anterior mandible three days before seeking medical attention at our department. Before he visited our hospital, his weight loss was unremarkable, and there was no change in his defecation habits. He had mild general malaise. He had no history of any major illnesses to date. The patient has a history of smoking 20 cigarettes per day for over 30 years, with no history of alcohol consumption.

**Figure 1:**
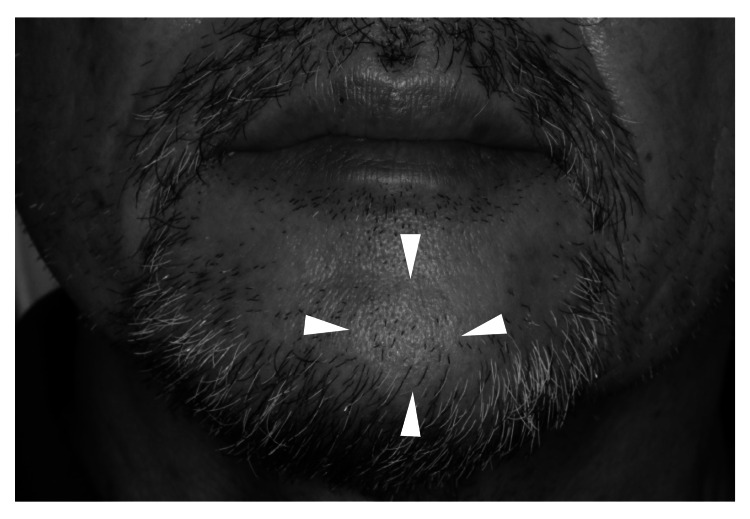
Extra-oral findings A pedicled mass was palpated on the left side of the chin.

A physical examination revealed no fever; he presented with a muscular build, poor nutritional status, and no signs of anemia in the eyelid conjunctiva or jaundice in the ocular conjunctiva. The facial appearance was symmetrical, but there was swelling of the size of the thumb tip observed on the left chin (Figure 1).

Examination revealed soybean-sized, elastic, soft, mobile, and tender bilateral submandibular lymph nodes, along with palpable mass formation in the left condyle and right mental region hypoesthesia. Intraoral findings included impaired mouth opening, mild redness of the buccal gingiva corresponding to 43 and 45, and percussion pain in the left mandible. Blood tests indicated a slight inflammatory reaction and subsequent tests revealed slightly elevated levels of lactate dehydrogenase (LDH) and alkaline phosphatase (ALP) (Table 1).

**Table 1:** Blood tests Blood tests indicated a slight inflammatory reaction and subsequent tests revealed slightly elevated levels of LDH and ALP. WBC: white blood cells; Neut: neutrophil; RBC: red blood cells; Plt: platelet; Hb: hemoglobin; CRP: C-reactive protein; AST: aspartate aminotransaminase; ALT: alanine aminotransferase; LDH: lactate dehydrogenase; ALP: alkaline phosphatase; TP: total protein; GT: glutamyl transpeptidase; BUN: blood urea nitrogen; FDP: fibrin degradation product

Test	Results	Unit	Normal Range
WBC	10060	/μL	3300-8600
Neut	57.3	%	38-80
RBC	502	10^4/μL	435-555
Plt	326	10^3/μL	158-348
Hb	15.4	g/dL	13.7-16.8
CRP	0.68	mg/dL	<0.14
AST	25	U/L	13-30
ALT	23	U/L	10-42
LDH	238	U/L	124-222
ALP	261	U/L	38-113
TP	6.6	g/dL	6.6-8.1
GT	152	U/L	13-64
BUN	20.6	mg/dL	8-20
FDP-P	15.4	μg/mL	<5
IgG	994	mg/dL	870-1700
IgA	230	mg/dL	110-410
IgM	93	mg/dL	33-190

**Figure 2:**
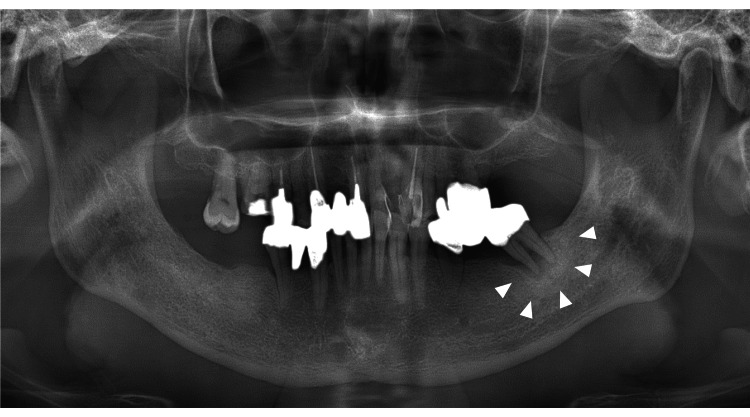
Panoramic radiograph Diffuse osteosclerosis equivalent to 37 was observed, and horizontal bone resorption of alveolar bone in the remaining tooth was moderate.

**Figure 3:**
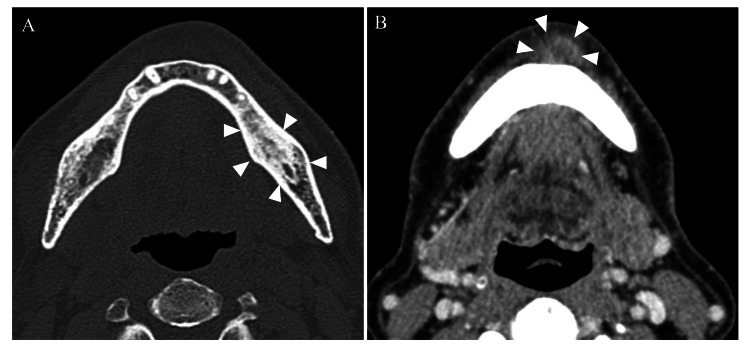
Contrast-enhanced CT photograph A: bone mode; B: contrast-enhanced mode Osteosclerosis was observed in the left side of the mandible, equivalent to 37, and a relatively well-defined, oval-shaped area showing a contrast effect at the margins was observed in the left side of the mandible.

Imaging findings included diffuse osteosclerosis in the panoramic X-ray, moderate horizontal bone resorption in the alveolar bone (Figure 2), and contrast-enhanced CT showing diffuse sclerosis around the root apex of tooth 37. A relatively well-defined, oval-shaped area showing a contrast effect at the margins was observed on the left side of the mandible (Figure 3).

**Figure 4:**
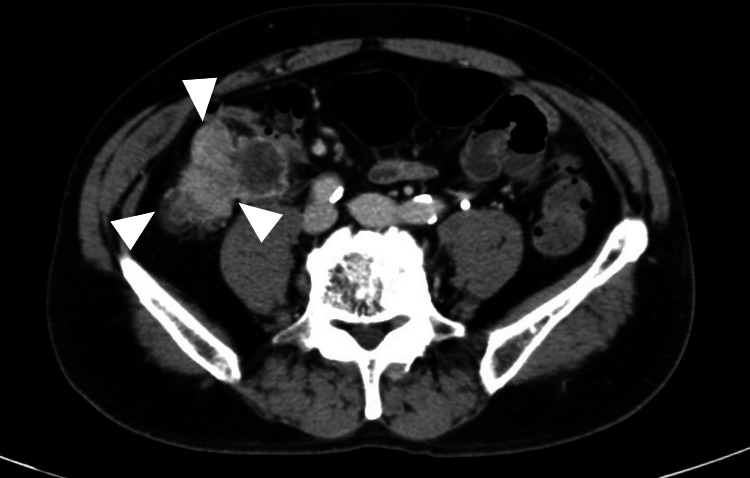
Contrast-enhanced CT of the abdomen Tumor formation with contrast effect was observed in the ileocecal region.

**Figure 5:**
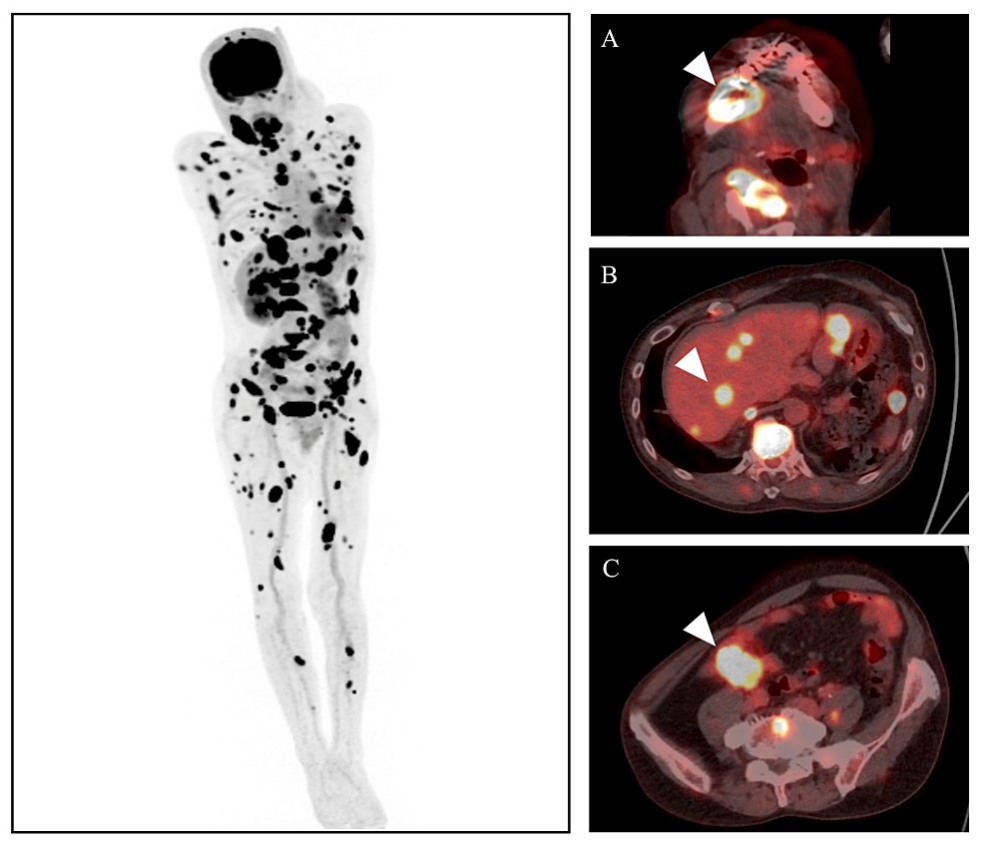
PET-CT image A: mandible; B: liver; C: colon F-18 fluorodeoxyglucose (FDG) was accumulated in the right mandible, multiple bone sites, liver, and colon.

The clinical diagnosis was bilateral mandibular osteomyelitis and numb chin syndrome. The patient was started on antimicrobial therapy with amoxicillin, but due to a skin rash, he was switched to clindamycin on day seven. Despite treatment, symptoms did not improve, and a tissue biopsy was offered but refused by the patient. On day 17, suspected NCS due to blood disease was suspected, and hematological investigations were initiated, showing no abnormalities. However, the patient experienced increased neck pain, leading to bone scintigraphy revealing abnormal accumulation in the whole body on day 28. Finally, we suspected the involvement of solid tumors, and blood tests were performed. CEA and CA19-9 levels were elevated on day 29. The patient was diagnosed with unresectable ileal colorectal cancer (cT4N1aMc2 Stage IVc) on day 31, confirmed by contrast-enhanced CT (Figure 4) and PET-CT (Figure 5).

The patient was admitted to the Department of Gastroenterology on day 44, and a diagnosis of adenocarcinoma was made by a partial biopsy using a lower gastrointestinal endoscope. After 10 months of systemic chemotherapy with FOLFOX+BEV, FOLFOXIRI, BEACON, and cetuximab, the patient is now in palliative care.

## Discussion

Numb chin syndrome refers to a sensory disturbance of the inferior alveolar nerve and miter nerve innervation and was first reported by Bell in 1829 and by Vincent et al. in 1896 [6,7]. Finally, in 1963, Calverley et al. reported five cases of metastatic malignancy of the mandible, which they termed numb chin syndrome [8]. NCS is caused by nerve invasion due to dental treatment or dental infection, or by compression or invasion of nerves due to cysts or neoplastic disease in the mandible. NCS can also be caused by hematological diseases such as leukemia and malignant lymphoma and the mandibular metastasis of solid tumors such as breast, lung, and prostate cancer. Babikir et al. reported that NCS is associated with solid tumor metastasis or hematologic disease in 30-50% of cases, and rare cases include mandibular metastasis of neuroendocrine tumors and lacunar infarctions [9,10]. Colorectal cancer has been a major cause of morbidity and mortality worldwide in recent years. Colorectal cancer is a malignancy that has seen an increase in both patients and deaths in recent years. Compared to the large number of patients with colorectal cancer, bone metastases are less common than in lung, breast, and kidney cancers, and metastases to the head and neck are rare [11]. NCS is the initial presentation, and to the best of our knowledge, there are no reports of multiple metastases of colorectal cancer.

Recently, with the increase in medication-related osteonecrosis of the jaw (MRONJ), it may be difficult to determine whether NCS is due to inflammation or metastasis of a malignant tumor, and Fortunato et al. retrospectively studied patients with NCS as the initial manifestation without definite dental or systemic factors. The authors found that the final diagnosis of MRONJ was made in 44.8% of the patients, and 48.3% of the patients had malignant disease as a cause. The authors also stated that most of the reported cases of NCS caused by malignant disease died two to three years later, that the appearance of NCS without a dental cause is a warning sign, that the differential diagnosis between malignant disease and MRONJ is difficult to distinguish between clinical symptoms, and that a biopsy is useful as a differential test in the early stages [12]. Lossos et al. suggest that patients with NCS with no apparent cause should be considered to have malignant disease until it is proven that malignant disease is not a factor [13].

In the present case, because of the initial lack of systemic symptoms and the unlikely possibility of malignant disease, additional imaging and blood tests could not be performed, and a month was required from the initial visit. The lack of symptoms of the primary disease in this study may be because the primary site was the ascending colon, which is usually recognized as the initial symptom of colorectal cancer, and therefore changes in defecation habits, bloody stools, abdominal pain, etc. were unlikely to occur. Furthermore, bone metastases from colorectal cancer often have a mixed pattern of metastasis, but in this case, there were no obvious osteolytic or osteoblastic changes, and there were few bone changes on contrast-enhanced CT, which also contributed to the difficulty in diagnosis. Although there was an episode of patient refusal to undergo biopsy, it was considered necessary to proceed with the biopsy strongly. The difficulty of obtaining imaging studies to evaluate the whole body in dentistry was another reason for the time required for diagnosis, and early consultation with an appropriate physician is necessary because of the possibility of malignant disease in NCS.

## Conclusions

We experienced a case of colorectal cancer with multiple metastases that metastasized to the mandible, with NCS as the initial manifestation. Dentists must always keep in mind that NCS can sometimes involve malignancy when dental treatment is not considered the cause. In this case, the patient refused a biopsy, so it took some time to make a diagnosis. However, when abnormal pain or dysesthesia is present, a tissue examination should be strongly recommended for the patient.
